# Neural Mechanisms Related to the Enhanced Auditory Selective Attention Following Neurofeedback Training: Focusing on Cortical Oscillations

**DOI:** 10.3390/app13148499

**Published:** 2023-07-23

**Authors:** Hwan Shim, Leah Gibbs, Karsyn Rush, Jusung Ham, Subong Kim, Sungyoung Kim, Inyong Choi

**Affiliations:** 1 Department of Electrical and Computer Engineering Technology, Rochester Institute of Technology, Rochester, NY 14623, USA;; 2 Department of Communication Sciences and Disorders, University of Iowa, Iowa City, IA 52242, USA;; 3 Department of Communication Sciences and Disorders, Montclair State University, Montclair, NJ 07043, USA;; 4 Graduate School of Culture Technology, Korea Advanced Institute of Science and Technology, Daejeon 34141, Republic of Korea; 5 Graduate School of Convergence Science and Technology, Seoul National University, Seoul 08826, Republic of Korea

**Keywords:** selective attention, auditory modulation, neural oscillation

## Abstract

Selective attention can be a useful tactic for speech-in-noise (SiN) interpretation as it strengthens cortical responses to attended sensory inputs while suppressing others. This cortical process is referred to as attentional modulation. Our earlier study showed that a neurofeedback training paradigm was effective for improving the attentional modulation of cortical auditory evoked responses. However, it was unclear how such neurofeedback training improved attentional modulation. This paper attempts to unveil what neural mechanisms underlie strengthened auditory selective attention during the neurofeedback training paradigm. Our EEG time–frequency analysis found that, when spatial auditory attention was focused, a fronto-parietal brain network was activated. Additionally, the neurofeedback training increased beta oscillation, which may imply top-down processing was used to anticipate the sound to be attended selectively with prior information. When the subjects were attending to the sound from the right, they exhibited more alpha oscillation in the right parietal cortex during the final session compared to the first, indicating improved spatial inhibitory processing to suppress sounds from the left. After the four-week training period, the temporal cortex exhibited improved attentional modulation of beta oscillation. This suggests strengthened neural activity to predict the target. Moreover, there was an improvement in the strength of attentional modulation on cortical evoked responses to sounds. The Placebo Group, who experienced similar attention training with the exception that feedback was based simply on behavioral accuracy, did not experience these training effects. These findings demonstrate how neurofeedback training effectively improves the neural mechanisms underlying auditory selective attention.

## 1. Introduction

Actively managing conversations in social interactions, known as “speech in noise (SiN)” understanding, recruits selective attention: enhancement of target speech and inhibition of noise. Large variance in understanding SiN is observed, even for individuals with normal hearing [1,2]. Poor understanding of SiN may be related to a decline in selective attention, due to the role of selective attention in enhancing target speech and inhibiting noise [3]. According to our recent finding [4], attentional modulation on neural encoding of acoustic inputs in the auditory cortex (AC) would be a crucial neural mechanism for successful SiN understanding. This finding showed that the amplitude ratio of auditory cortical responses to the target speech and noise during a SiN task correlated with behavioral SiN performance [5–8].

Recent studies [9,10] demonstrated that training on maintaining attention to low-intensity signals extracted from background noise improved SiN performance and was transferable to untrained stimuli. In these studies, both auditory segregation and selective attention were involved in the training effects. To explicitly reinforce attentional modulation of cortical auditory evoked responses, our previous study created a neurofeedback training paradigm [11]. Two voice streams—spoken by a high-pitched and a low-pitched speaker—were played from different directions (left and right) with no physical overlap in time (i.e., no energetic masking) to maximize stream segregation. Subjects were tasked with focusing on one voice stream at a time. Throughout this auditory selective attention training, 64-channel electroencephalography (EEG) decoded auditory selective attention from single-trial EEG signals [12–14]. Only the Experimental Group participants received visual feedback based on the EEG-based attention decoder. The Control Group went through a comparable selective attention training program without receiving neurofeedback to rule out a placebo effect. The accuracy of attention decoding from single-trial EEG signals reflects the strength of attentional modulation on cortical auditory evoked responses [13]. Therefore, giving users the outcome of EEG-based attention decoding in the form of neurofeedback [15] may strengthen their attentional modulation of cortical response.

However, it is still elusive which neural mechanisms enable strengthened auditory selective attention and which crucial auditory cues drive the training effect in the neurofeedback training paradigm, where the competing streams varied in location, speaker identity, and tempo to maximize stream segregation. This poses the question of how to reveal what neural mechanisms and auditory cues explicitly reinforce attentional modulation of cortical auditory evoked responses. The present study utilized the induced oscillation activity to investigate this. More specifically, this paper provides further analyses of our previous data [11] focusing on how cortical oscillations changed over the course of repeated training.

A fronto-parietal brain network is activated when spatial auditory attention is focused. Attention to spatial auditory features leads to increased fronto-parietal network activity in functional magnetic resonance imaging (fMRI; [16–20]), electroencephalography (EEG; [21]), and magnetoencephalography (MEG; [22]). Similar to visual spatial attention, auditory spatial attention causes parietal lateralization of alpha oscillation power in the intraparietal sulcus (IPS; [21,23–25]). Alpha lateralization varies systematically when attentional focus changes, similar to vision [26]. Alpha lateralization is more transient if non-spatial characteristics encourage stream segregation, but it is persistent when competing streams are comparable aside from spatial attributes [23].

Spatial signals are nevertheless an efficient means for focusing top-down attention while having a weak role in auditory grouping and streaming [27–29]. Both bottom-up auditory cues and top-down cognitive processes are used when listening to speech in noisy environments [30]. There are low-level oscillatory cortical mechanisms that use sensory sampling or prediction while maintaining different neural rhythms based on the task [31]. As previous work supports, the beta wave employs top-down processing to transmit descending information [32]. The brain relies extensively on predictions and expectations to fill in the blanks, thus employing prior information.

Therefore, we hypothesize that while a subject actively recruits selective attention during the task, the alpha band oscillation would be present in the IPS, and the beta band oscillation would be located in the middle temporal gyrus (MTG), superior temporal sulcus (STS), and inferior frontal gyrus (IFG) during the preparatory period. The preparatory period represents the time interval between the presentation of the auditory cue and the target speech with the masker, during which a cognitive process to steer the listeners’ selective attention is expected to emerge.

## 2. Materials and Methods

This study utilized data from [11]. As such, most methods are consistent with the previous study.

### 2.1. Participants

A total of 20 native American English speakers with normal hearing thresholds were recruited from the University of Iowa student population (mean age: 23.2 years, SD: 1.33 years, 6 males: 30%). All the participants had 20 dB HL or lower thresholds at any tested frequency, measured in octaves from 250 to 8000 Hz. After consenting to participate in the trial, participants were randomized to either the Experimental or Placebo Group (i.e., single-blinded design). All participants underwent four consecutive weeks of training lasting one hour per week, as well as pre- and post-training speech-in-noise (SiN) tests at their first and last visits. The work was finished in compliance with the International Medical Association’s Code of Ethics, and written informed consent was acquired (Declaration of Helsinki). The Institutional Review Board at the University of Iowa examined and approved all study methods [11].

A power analysis based on the effect size reported by earlier perceptual training studies, such as [9], can support the sample size. These estimates indicated that 10 people per group would be needed for the current study, assuming a significance level of 0.05 and a power of 0.80. We chose a 4-week training period, as did [9], in order to (1) ensure overnight consolidation, which has been claimed to be essential for perceptual training [33–35] and (2) prevent learning and memory of speech stimuli that were used in pre- and post-training tests [36,37].

### 2.2. Task Design and Procedures

#### 2.2.1. Attention Training Procedure: Experimental Group

**Figure 1.**
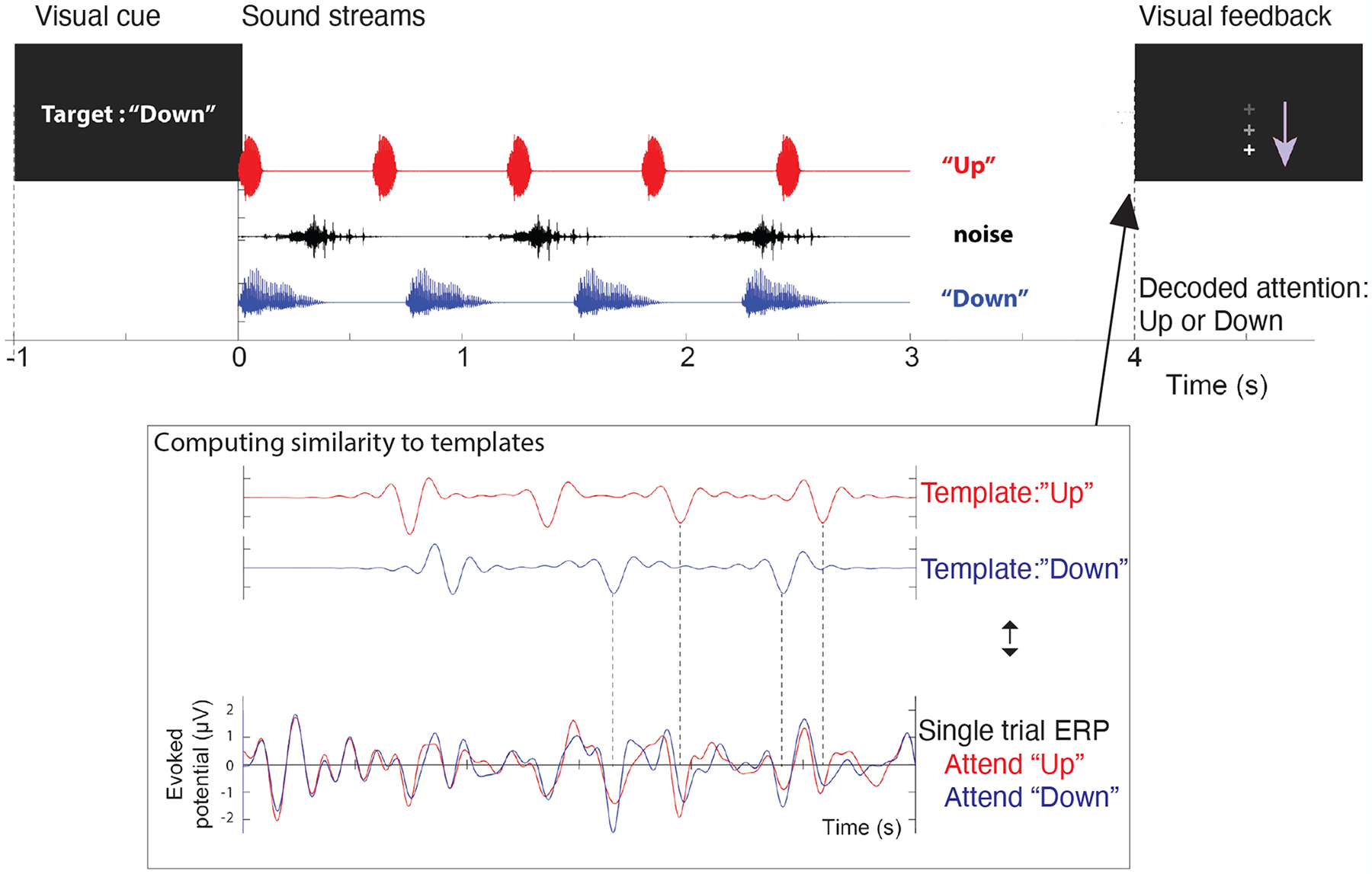
An example of the neurofeedback training’s trial structure assigned to the Experimental Group. An attend-*down* case is shown [11].

Three overlapping auditory streams were presented during each training session: a male voice saying the word “down” four times from the right (+30 azimuth) loudspeaker, a female voice saying the word “up” five times from the left (−30 azimuth), and an unrelated distractor noise that sounds like a water splash played three times intermittently from the loudspeaker directly in front of the subject. The presentation level was 70 dB SPL calibrated using an SPL meter which means the experiment’s auditory stimulus was played at a volume that led the SPL meter to indicate 70 dB in the listening position, and this level was verified each time the experiment was performed. A visual cue (“Target: Up” or “Target: Down”) was used for each of the 120 trials during each visit to direct participants’ attention to the “up” or “down” stream (60 trials each). The attended stream was decoded from the EEG after the stimuli were shown. After each trial, visual feedback (“+” sign on the screen going up or down) was presented to show the decoded direction of attention (i.e., whether the “up” or “down” stream” was attended). An example of a trial going “down” stream is shown in Figure 1 [11].

#### 2.2.2. Attention Training Procedure: Placebo Group

The Placebo Group heard the similar three overlapping auditory streams with the exception that one of the last three (for the “up” stream) or two (for the “down” stream) utterances in each stream had a three-semitone higher pitch. These streams consisted of isochronous repetitions of “up” and “down” spoken by the female and male speakers with a distractor noise. They chose the utterance with a higher pitch in the attended stream by hitting the number key as the visual cue directed their attention to either the “up” or “down” stream in each trial (i.e., an oddball detection task within a trial). Depending on the correctness of their button response, they received visible feedback (“Correct” or “Incorrect”) after pressing the button [11].

### 2.3. Induced Oscillatory Activity Analysis

Using the BioSemi ActiveTwo system, 64 channels of scalp EEG data were captured throughout the training tasks at a sampling rate of 2048 Hz using the international 10–20 configuration. For the induced oscillatory activity analysis, 64-channel EEG data from 5 subjects in the Experimental Group and 10 subjects in the Placebo Group were collected and analyzed. EEG data from the other 5 subjects in the Experimental could not be used since the full 64-channel single-trial data were not saved for later off-line analyses in the version used for those subjects, while the behavioral and single-trial EEG classification analysis could be applied to all the 20 subjects.

A template-matching approach was utilized to decode the attended stream from single-trial EEG signals and deliver neurofeedback to the Experimental Group [13]. EEG recordings from linked mastoids were averaged and re-referenced to the front-central channels (Fz, FCz, FC1, FC2, and Cz). After baseline correction and bandpass filtering between 1 and 9 Hz, EEG data were compared to two template waveforms created from grand-average cortical evoked responses to the single “up” and “down” streams while passive listening in silence. The attention was decoded by choosing the template that has a larger correlation coefficient with the single-trial EEG data. The fixation cross was then moved upward or downward on the computer screen to provide visual feedback in response to the detection of attention on the “up” stream or “down” stream, respectively.

The spectrograms were processed from the resampled EEG signal per channel and trial based on a 256-sample fast Fourier transform with a period of 64 samples and an overlap of 63 samples in 256 Hz sampling rate. These spectrograms were categorized into “up” or “down”, and weeks. The paired t-tests between “up” and “down” every week were taken. Furthermore, the paired t-test between “up”–“down” in week 4 and “up”–“down” in week 1 were taken to explore the difference in the attentional modulation. In addition, the cluster-based permutation analysis was performed to find the significantly different clusters of attentional modulation between week 4 and week 1 for the Experimental Group and the Placebo Group [38]. To observe the difference between week 4 and week 1 in each alpha and beta oscillation, we selected frequency bands for each alpha and beta range (8–13 Hz and 24–29 Hz, respectively), set the minimum cluster length as around 50 ms, and set the p-values for the t-test as 0.05 in the spectrograms. For the Experimental Group and the Placebo Group, the cluster-based permutation analysis was performed separately in each alpha and beta band and the obtained clusters implied the statistically significant difference between week 4 and week 1 for each group.

Source localization is needed to analyze temporal dynamics with the estimated source spatial distribution because sensor data in some channels may not sufficiently capture the geographic distribution of neural sources and the temporal dynamics of ERP components [39]. The inverse operator was estimated using minimum norm estimation (MNE) [40–42] based on assumptions of multiple sparse priors [43] on an average template brain in order to project the sensor space data into source space. By using the inverse operator, source space time courses of ERPs were acquired over all cortical voxels in both hemispheres. As a method of noise normalization, they were projected onto the cortical maps to create dynamic statistical parametric maps (dSPMs) [44]. Each cluster for beta and alpha waves in the preparatory period was selected and all voxels were averaged during each selected cluster for beta and alpha waves. The timely averaged voxels were presented as snapshots of the clusters for the Experimental Group and the Placebo Group.

## 3. Results

### 3.1. Enhanced Attentional Modulation

A single-trial EEG waveform and the grand-average cortical evoked responses to the single “up” and “down” streams were compared using Pearson correlation coefficients to decode selective attention. With continued training, attentional modulation altered over time. In the Experimental Group, the mean decoding accuracy increased monotonically from 55.9% (SD = 4.1%) in the first week to 57.4% (SD = 6.2%) in the second week, 58.0% (SD = 3.8%) in the third week, and 60.2% (SD = 3.7%) in the fourth week. The Placebo Group, on the other hand, did not exhibit a progression in attentional modulation with time. For the first, second, third, and most recent weeks, the mean decoding accuracies were 60.7, 59.7, 54.1, and 58.9%, respectively. The relative standard deviations were 7.0, 3.5, 5.0, and 3.4% [11].

A two-way mixed ANOVA on the decoding accuracy observed in the first and last week was conducted to further investigate the effect of training time (first vs. fourth week), the type of feedback (neurofeedback vs. behavioral), and the interaction of those effects on the attentional modulation (i.e., quantified as the decoding accuracy). There were no significant main effects of time (F_1,18_ = 0.99, *p* = 0.33) or group (F_1,18_ = 1.0, *p* = 0.32) on decoding accuracy, indicating that (1) there was no baseline difference in attentional modulation between the groups and (2) there was no significant improvement in attentional modulation over time with repeated training. However, the results showed a significant interaction between time and group (F_1,18_ = 5.7, *p* = 0.028), showing that the effects of training on attentional modulation over time varied significantly between the groups. In the Experimental Group, the post hoc paired t-test between decoding accuracy in the first and last weeks showed a Bonferroni corrected *p*-value of 0.022 (six times the uncorrected *p*-value of 0.0036) [11].

### 3.2. Induced Cortical Activity Changes in Source Space Topography to Selective Attention

Examining the ERPs acquired at the sensor space was the initial step in determining the EEG data’s quality. The induced cortical activity from the EEG data was able to be assessed due to the clear auditory components (like N1) discovered from the front-central channels.

**Figure 2.**
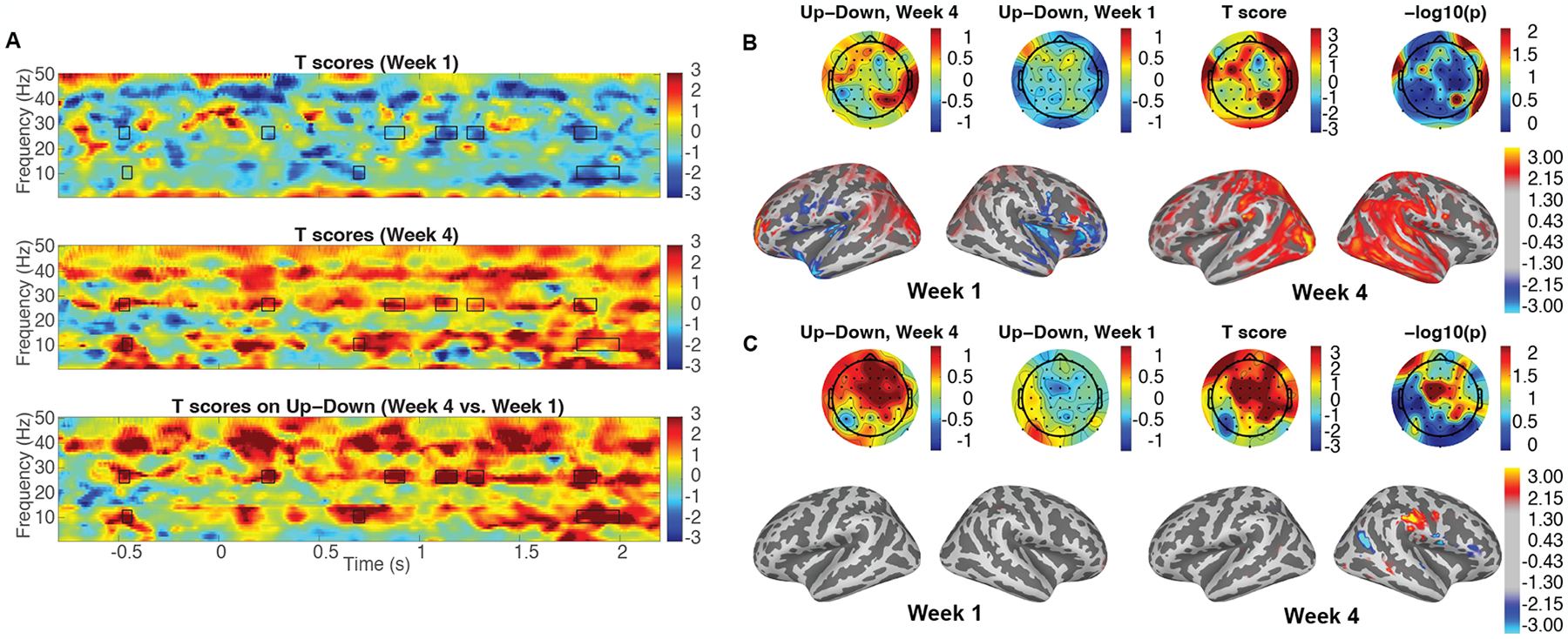
Spectrograms and topographies of induced activities in the Experimental Group. (**A**) The t-score spectrograms of auditory modulation between “Up” and “Down” in week 1, week 4 and the t-score spectrogram of “Up” vs. “Down” between week 4 and week 1 are obtained during the neurofeedback training from the Experimental Group. The solid-line boxes indicate significantly different clusters within the alpha and beta wave ranges (8–13 Hz and 24–29 Hz, respectively), obtained by the cluster-based permutation tests from the Experimental Group [38]. (**B**) The topographies of the beta wave cluster are obtained in the preparatory time range before target speech onset (from −0.50 to −0.45 s) at the sensor space and source space. (**C**) The topographies of the alpha wave cluster are obtained in the preparatory time range before target speech onset (from *−*0.48 to −0.43 s) at the sensor space and source space.

**Figure 3.**
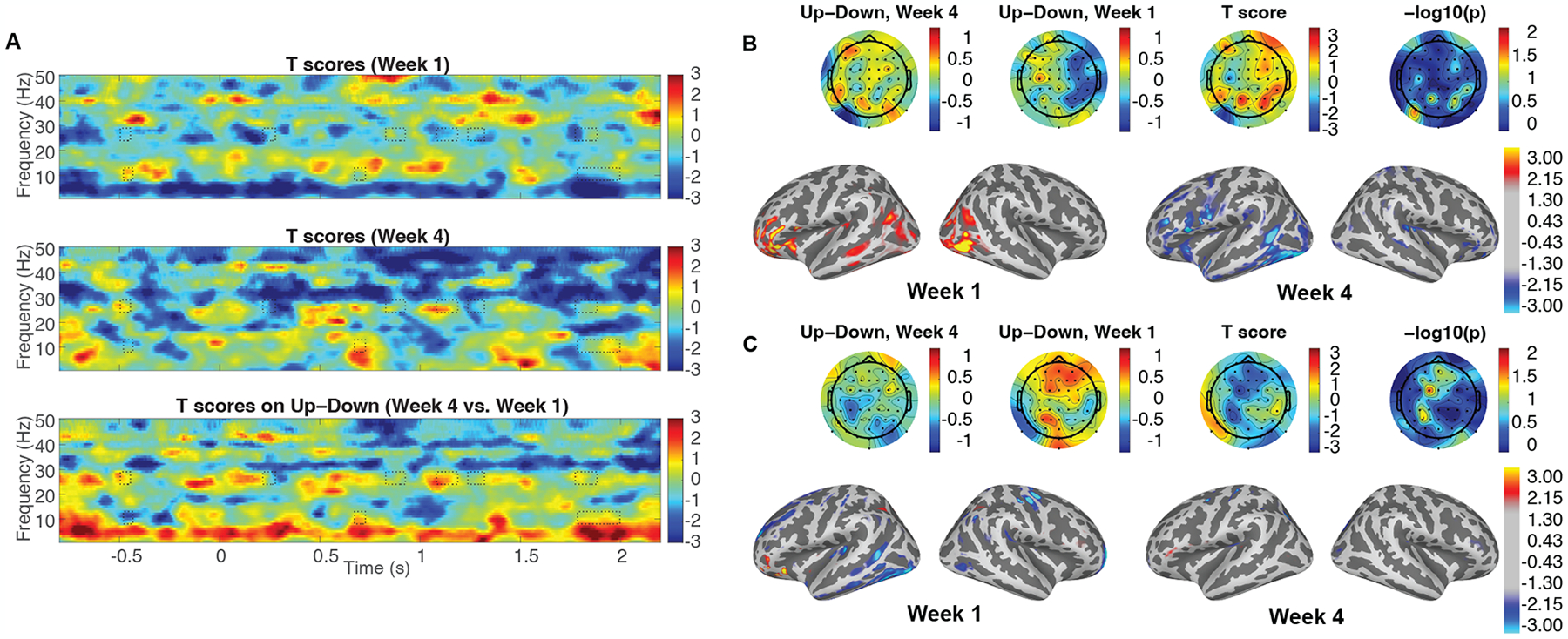
Spectrograms and topographies of induced activities in the Placebo Group. (**A**) The t-score spectrograms of auditory modulation between “Up” and “Down” in week 1, week 4, and between week 4 and week 1 are obtained during the neurofeedback training from the Placebo Group. No significantly different clusters are found in the Placebo Group. To indicate where the regions of interest are, the dotted line boxes indicate significantly different clusters within the alpha and beta wave ranges (8–13 Hz and 24–29 Hz, respectively), obtained by the cluster-based permutation tests from the Experimental Group [38]. (**B**) The mean topographies of the beta wave cluster taken from the Placebo Group are obtained in the preparatory time range before target speech onset (from −0.50 to −0.45 s) at the sensor space and source space. (**C**) The topographies of the alpha wave cluster taken from the Placebo Group are obtained in the preparatory time range before target speech onset (from −0.48 to −0.43 s) at the sensor space and source space.

*T*-tests were taken between “Up” and “Down” in the spectrogram domain for week 1 and week 4, and of “Up”–“Down” in the spectrogram between week 4 and week 1 to analyze the difference of auditory modulation for both the Experimental Group and the Placebo Group. The cluster-based permutation tests were performed in the spectrogram which included the difference of attentional modulation between week 4 and week 1. Significantly different clusters were obtained within alpha and beta wave ranges (8–13 Hz and 24–29 Hz, respectively), in the Experimental Group, displayed as solid-line boxes in Figure 2A. These significant differences were not observed in the Placebo Group, so in order to indicate the regions of interest, the clusters from the Experimental Group were displayed as dotted line boxes in Figure 3A. As the clusters showed, attentional modulation was significantly improved only in the Experimental Group which received neurofeedback in every trial. When examining the alpha and beta waves in the preparatory timeline (from −1 to 0 s), each cluster appeared in the alpha and beta frequency ranges. The sensor space topographies and the source space topographies for both groups were averaged in the time–frequency range of the clusters from the Experimental Group, shown in Figure 2B,C and Figure 3B,C. The sensor space topographies of the beta oscillation within the cluster show a significantly stronger power on the parietal region of both hemispheres in week 4 of the Experimental Group. The source space topographies of the beta wave range indicate more power on MTG, STS, and IFG of both hemispheres in week 4 of the Experimental Group, illustrated in Figure 2B. The beta oscillations expand their role to anticipate selective attention on the given auditory cues during preparatory time periods in week 4 of the Experimental Group. Between week 1 and week 4 of the Placebo Group, there was no significant difference in the sensor space and source space topographies of the beta wave range during the preparatory time periods.

The sensor space topographies of the alpha wave range from the time periods of the cluster show a significantly strong power on the right parietal region in week 4 of the Experimental Group. The source space topographies of the alpha wave range show more power on IPS of the right hemisphere in week 4 of the Experimental Group in Figure 2C. The alpha oscillations increase to inhibit the left auditory stimuli during preparatory time periods in week 4 of the Experimental Group. During the preparatory time periods, there was no significant difference in the sensor space and source space topographies of the alpha wave range between week 1 and week 4 of the Placebo Group.

## 4. Discussion

### 4.1. Conclusions

The current study investigated what cortical mechanisms are involved when auditory selective attention is improved by perceptual training. For this purpose, we further analyzed our previous dataset [11] which demonstrated the efficacy of our neurofeedback training paradigm for improving auditory selective attention [4–11]. By examining induced cortical oscillations, in the Experimental Group (who received neurofeedback), we found that alpha (~10 Hz) and beta (~25 Hz) oscillations in the right parietal cortex increase over the course of training. The Placebo Group (who did not receive neurofeedback) did not show any significant change in cortical oscillations. There was no significant change in alpha and beta oscillations in the Placebo Group. These findings provide novel perspectives of the brain plasticity related to enhanced auditory selective attention following neurofeedback training, which was not provided by our previous report [11]. We claim that the stronger beta oscillation in the Experimental Group indicates more strongly engaged top-down processing to predict forth-coming sounds to attend in advance [45,46]. Beta oscillation is believed to be related to top-down prediction based on the context of stimuli [46,47]. Especially, enhancement in beta is observed during the tasks that require top-down attention [48,49]. Theves et al. [50] showed that for the audiovisual onset synchrony task, the preparatory beta oscillations in the central, parietal, and temporal lobes increased after training. Our result indicates that this is also true in more auditory-centered tasks. Our result provides more support with contrasting outcomes in the Placebo Group where no neurofeedback was given.

Likewise, the increase in alpha oscillation in the right parietal region can be interpreted as enhanced inhibitory processing to ignore competing sounds. According to Viswanathan et al. [51], single-trial speech intelligibility in speech-in-noise tasks considerably correlates with, and independently contributes to the overall magnitudes of alpha power in parieto-occipital EEG channels and beta power in frontal channels. Furthermore, Price et al. [52] demonstrated that accuracy in SiN is predicted by the alpha modulation between clean and noise-degraded speech. Similarly, in this study, SiN results were improved when alpha power modulation was shown across the training period in the Experimental Group, and SiN results were not improved when alpha power modulation was not shown across the training in the Placebo Group [11]. Moreover, as in Obleser and Weisz [53], the enhancement of alpha in the parietal region can be used as a predictor of the SiN results. The right parietal stimulation of alpha was observed, but rightward spatial attention was not enhanced, consistent with the results by Deng et al. [54]. It is also consistent with the finding by Frey et al. [55] that the parietal cortex is asymmetrical and alpha oscillation inhibits contralateral attention. In addition, as in the topography of Deng et al. [26], parieto-occipital alpha power during the attentional preparatory period appeared strongly on the right side despite attention to the left side. Additionally, the topographies were asymmetric and did not show a linear relationship with attention along the side from left to right. This could be because the left auditory cortex mainly localizes stimuli in the contralateral space, while the right auditory cortex processes stimuli in the whole space [55].

### 4.2. Limitations of the Study

There are several limitations of this study. First, the current experimental paradigm divided the Experimental Group and the Placebo Group depending on whether visual neurofeedback was given or not. A future study will also be conducted on how attentional modulation would change when neurofeedback is intentionally provided to participants as incorrect results by randomizing visual feedback.

The concurrent speech streams were disparate in location, speaker identity, and tempo in order to maximize stream separation. Both parietal alpha oscillation and temporal beta oscillation were observed during the preparatory period when the subjects were focused, and valid feedback was provided. This indicates both spatial and pitch-related mechanisms contributed. In order to specify key auditory cues that contribute to the training effect, future research should test the effectiveness of various auditory cues for selective attention training and determine whether these cues are also present in a more general SiN task.

Pearson correlation coefficients between the grand-average cortical evoked responses and a single-trial EEG waveform were used for decoding selective attention in this study. The intraclass correlation coefficients might be used for the decoder. For the follow-up study, it would be considered if the decoding algorithm is updated to improve the mean decoding accuracy by using machine learning algorithms. Also, the whole 64-channel EEG data from a smaller number of subjects in the Experimental Group were used for the induced activity analysis, and the small sample size in one group was a limitation of this study. A future study will recruit a greater number of subjects.

This study did not reveal whether the training impact endures after the training time or disappears. A follow-up study is anticipated to investigate this question. Lastly, because selective listening in background noise is hampered by peripheral hearing loss [56], additional research is required to examine the training effect in clinical populations (e.g., hearing aid or cochlear implant users [11]).

## Data Availability

The raw data supporting the conclusions of this article will be made available by the authors, without undue reservation.

## References

[1] Kumar G; Amen F; Roy D Normal Hearing Tests: Is a Further Appointment Really Necessary? J. R. Soc. Med 2007, 100, 66. 10.1258/jrsm.100.2.66-a PMC1791002 17277271

[2] Moore DR; Rosen S; Bamiou DE; Campbell NG; Sirimanna T; James Bellis T; Chermak G; Weihing J; Musiek F; Dillon H; Evolving Concepts of Developmental Auditory Processing Disorder (APD): A British Society of Audiology APD Special Interest Group “White Paper”. Int. J. Audiol 2013, 52, 3–13. 23039930 10.3109/14992027.2012.723143

[3] Bressler S; Goldberg H; Shinn-Cunningham B Sensory Coding and Cognitive Processing of Sound in Veterans with Blast Exposure. Hear. Res 2017, 349, 98. 27815131 10.1016/j.heares.2016.10.018 PMC5645017

[4] Kim S; Schwalje AT; Liu AS; Gander PE; McMurray B; Griffiths TD; Choi I Pre- and Post-Target Cortical Processes Predict Speech-in-Noise Performance. Neuroimage 2021, 228, 117699. 33387631 10.1016/j.neuroimage.2020.117699 PMC8291856

[5] Hillyard SA; Hink RF; Schwent VL; Picton TW Electrical Signs of Selective Attention in the Human Brain. Science 1973, 182, 177–180. 4730062 10.1126/science.182.4108.177

[6] Hillyard SA; Vogel EK; Luck SJ Sensory Gain Control (Amplification) as a Mechanism of Selective Attention: Electrophysiological and Neuroimaging Evidence. Philos. Trans. R. Soc. B Biol. Sci 1998, 353, 1257. 10.1098/rstb.1998.0281 PMC1692341 9770220

[7] Mesgarani N; Chang EF Selective Cortical Representation of Attended Speaker in Multi-Talker Speech Perception. Nature 2012, 485, 233–236. 22522927 10.1038/nature11020 PMC3870007

[8] Carcea I; Insanally MN; Froemke RC Dynamics of Auditory Cortical Activity during Behavioural Engagement and Auditory Perception. Nat. Commun 2017, 8, 14412. 28176787 10.1038/ncomms14412 PMC5309852

[9] Whitton JP; Hancock KE; Polley DB Immersive Audiomotor Game Play Enhances Neural and Perceptual Salience of Weak Signals in Noise. Proc. Natl. Acad. Sci. USA 2014, 111, E2606–E2615. 24927596 10.1073/pnas.1322184111 PMC4078866

[10] Whitton JP; Hancock KE; Shannon JM; Polley DB Audiomotor Perceptual Training Enhances Speech Intelligibility in Background Noise. Curr. Biol 2017, 27, 3237–3247.e6. 29056453 10.1016/j.cub.2017.09.014 PMC5997394

[11] Kim S; Emory C; Choi I Neurofeedback Training of Auditory Selective Attention Enhances Speech-In-Noise Perception. Front. Hum. Neurosci 2021, 15. 10.3389/fnhum.2021.676992 PMC8258151 34239430

[12] Kerlin JR; Shahin AJ; Miller LM Attentional Gain Control of Ongoing Cortical Speech Representations in a “Cocktail Party”. J. Neurosci 2010, 30, 620–628. 20071526 10.1523/JNEUROSCI.3631-09.2010 PMC2832933

[13] Choi I; Rajaram S; Varghese LA; Shinn-Cunningham BG Quantifying Attentional Modulation of Auditory-Evoked Cortical Responses from Single-Trial Electroencephalography. Front. Hum. Neurosci 2013, 7, 115. 23576968 10.3389/fnhum.2013.00115 PMC3616343

[14] O’Sullivan JA; Power AJ; Mesgarani N; Rajaram S; Foxe JJ; Shinn-Cunningham BG; Slaney M; Shamma SA; Lalor EC Attentional Selection in a Cocktail Party Environment Can Be Decoded from Single-Trial EEG. Cereb. Cortex 2015, 25, 1697–1706. 24429136 10.1093/cercor/bht355 PMC4481604

[15] Sherlin LH; Arns M; Lubar J; Heinrich H; Kerson C; Strehl U; Sterman MB Neurofeedback and Basic Learning Theory: Implications for Research and Practice. J. Neurother 2011, 15, 292–304.

[16] Alho K; Salmi J; Koistinen S; Salonen O; Rinne T Top-down Controlled and Bottom-up Triggered Orienting of Auditory Attention to Pitch Activate Overlapping Brain Networks. Brain Res 2015, 1626, 136–145. 25557401 10.1016/j.brainres.2014.12.050

[17] Hill KT; Miller LM Auditory Attentional Control and Selection during Cocktail Party Listening. Cereb. Cortex 2010, 20, 583–590. 19574393 10.1093/cercor/bhp124 PMC2820699

[18] Kong L; Michalka SW; Rosen ML; Sheremata SL; Swisher JD; Shinn-Cunningham BG; Somers DC Auditory Spatial Attention Representations in the Human Cerebral Cortex. Cereb. Cortex 2014, 24, 773–784. 23180753 10.1093/cercor/bhs359 PMC3920769

[19] Michalka SW; Kong L; Rosen ML; Shinn-Cunningham BG; Somers DC Short-Term Memory for Space and Time Flexibly Recruit Complementary Sensory-Biased Frontal Lobe Attention Networks. Neuron 2015, 87, 882–892. 26291168 10.1016/j.neuron.2015.07.028 PMC4545499

[20] Noyce AL; Cestero N; Michalka SW; Shinn-Cunningham BG; Somers DC Sensory-Biased and Multiple-Demand Processing in Human Lateral Frontal Cortex. J. Neurosci 2017, 37, 8755–8766. 28821668 10.1523/JNEUROSCI.0660-17.2017 PMC5588466

[21] Banerjee S; Snyder AC; Molholm S; Foxe JJ Oscillatory Alpha-Band Mechanisms and the Deployment of Spatial Attention to Anticipated Auditory and Visual Target Locations: Supramodal or Sensory-Specific Control Mechanisms? J. Neurosci 2011, 31, 9923–9932. 21734284 10.1523/JNEUROSCI.4660-10.2011 PMC3343376

[22] Huang S; Chang W-T; Belliveau JW; Hämäläinen M; Ahveninen J Lateralized Parietotemporal Oscillatory Phase Synchronization during Auditory Selective Attention. Neuroimage 2014, 86, 461–469. 24185023 10.1016/j.neuroimage.2013.10.043 PMC3908553

[23] Bonacci LM; Bressler S; Shinn-Cunningham BG Nonspatial Features Reduce the Reliance on Sustained Spatial Auditory Attention. Ear Hear 2020, 41, 1635–1647. 33136638 10.1097/AUD.0000000000000879 PMC9831360

[24] Bonacci LM; Dai L; Shinn-Cunningham BG Weak Neural Signatures of Spatial Selective Auditory Attention in Hearing-Impaired Listeners. J. Acoust. Soc. Am 2019, 146, 2577. 31671991 10.1121/1.5129055 PMC7273515

[25] Tune S; Wöstmann M; Obleser J Probing the Limits of Alpha Power Lateralisation as a Neural Marker of Selective Attention in Middle-Aged and Older Listeners. Eur. J. Neurosci 2018, 48, 2537–2550. 29430736 10.1111/ejn.13862

[26] Deng Y; Choi I; Shinn-Cunningham B Topographic Specificity of Alpha Power during Auditory Spatial Attention. Neuroimage 2020, 207, 116360. 31760150 10.1016/j.neuroimage.2019.116360 PMC9883080

[27] Darwin CJ; Hukin RW Auditory Objects of Attention: The Role of Interaural Time Differences. J. Exp. Psychol. Hum. Percept. Perform 1999, 25, 617–629. 10385981 10.1037//0096-1523.25.3.617

[28] Sach AJ; Bailey PJ Some Characteristics of Auditory Spatial Attention Revealed Using Rhythmic Masking Release. Percept. Psychophys 2004, 66, 1379–1387. 15813201 10.3758/bf03195005

[29] Shinn-Cunningham BG Object-Based Auditory and Visual Attention. Trends Cogn. Sci 2008, 12, 182–186. 18396091 10.1016/j.tics.2008.02.003 PMC2699558

[30] Strait DL; Kraus N Can You Hear Me Now? Musical Training Shapes Functional Brain Networks for Selective Auditory Attention and Hearing Speech in Noise. Front. Psychol 2011, 2, 113. 21716636 10.3389/fpsyg.2011.00113 PMC3115514

[31] Arnal LH; Giraud AL Cortical Oscillations and Sensory Predictions. Trends Cogn. Sci 2012, 16, 390–398. 22682813 10.1016/j.tics.2012.05.003

[32] Wang XJ Neurophysiological and Computational Principles of Cortical Rhythms in Cognition. Physiol. Rev 2010, 90, 1195–1268. 20664082 10.1152/physrev.00035.2008 PMC2923921

[33] Fenn KM; Nusbaum HC; Margoliash D Consolidation during Sleep of Perceptual Learning of Spoken Language. Nature 2003, 425, 614–616. 14534586 10.1038/nature01951

[34] Eisner F; McQueen JM Perceptual Learning in Speech: Stability over Time. J. Acoust. Soc. Am 2006, 119, 1950. 16642808 10.1121/1.2178721

[35] Davis MH; Di Betta AM; Macdonald MJE; Gaskell MG Learning and Consolidation of Novel Spoken Words. J. Cogn. Neurosci 2009, 21, 803–820. 18578598 10.1162/jocn.2009.21059 PMC2832742

[36] Bentler RA List Equivalency and Test-Retest Reliability of the Speech in Noise Test. Am. J. Audiol 2000, 9, 84–100. 11200196 10.1044/1059-0889(2000/010)

[37] Booshardt S; Degonda N; Schmidt CF; Boesiger P; Nitsch RM; Hock C; Henke K One Month of Human Memory Consolidation Enhances Retrieval-Related Hippocampal Activity. Hippocampus 2005, 15, 1026–1040. 16015623 10.1002/hipo.20105

[38] Maris E; Oostenveld R Nonparametric Statistical Testing of EEG- and MEG-Data. J. Neurosci. Methods 2007, 164, 177–190. 17517438 10.1016/j.jneumeth.2007.03.024

[39] Tian X; Huber DE Measures of Spatial Similarity and Response Magnitude in MEG and Scalp EEG. Brain Topogr 2008, 20, 131–141. 18080180 10.1007/s10548-007-0040-3

[40] Hämäläinen MS; Sarvas J Realistic Conductivity Geometry Model of the Human Head for Interpretation of Neuromagnetic Data. IEEE Trans. Biomed. Eng 1989, 36, 165–171. 2917762 10.1109/10.16463

[41] Gramfort A; Luessi M; Larson E; Engemann DA; Strohmeier D; Brodbeck C; Parkkonen L; Hämäläinen MS MNE Software for Processing MEG and EEG Data. Neuroimage 2014, 86, 446–460. 24161808 10.1016/j.neuroimage.2013.10.027 PMC3930851

[42] Gramfort A; Luessi M; Larson E; Engemann DA; Strohmeier D; Brodbeck C; Goj R; Jas M; Brooks T; Parkkonen L; MEG and EEG Data Analysis with MNE-Python. Front. Neurosci 2013, 7, 267. 24431986 10.3389/fnins.2013.00267 PMC3872725

[43] Friston K; Harrison L; Daunizeau J; Kiebel S; Phillips C; Trujillo-Barreto N; Henson R; Flandin G; Mattout J Multiple Sparse Priors for the M/EEG Inverse Problem. Neuroimage 2008, 39, 1104–1120. 17997111 10.1016/j.neuroimage.2007.09.048

[44] Dale AM; Liu AK; Fischl BR; Buckner RL; Belliveau JW; Lewine JD; Halgren E Dynamic Statistical Parametric Mapping: Combining FMRI and MEG for High-Resolution Imaging of Cortical Activity. Neuron 2000, 26, 55–67. 10798392 10.1016/s0896-6273(00)81138-1

[45] Engel AK; Fries P; Singer W Dynamic Predictions: Oscillations and Synchrony in Top-down Processing. Nat. Rev. Neurosci 2001, 2, 704–716. 11584308 10.1038/35094565

[46] Bressler SL; Richter CG Interareal Oscillatory Synchronization in Top-down Neocortical Processing. Curr. Opin. Neurobiol 2015, 31, 62–66. 25217807 10.1016/j.conb.2014.08.010

[47] Engel AK; Fries P Beta-Band Oscillations—Signalling the Status Quo? Curr. Opin. Neurobiol 2010, 20, 156–165. 20359884 10.1016/j.conb.2010.02.015

[48] Riddle J; Hwang K; Cellier D; Dhanani S; D’esposito M Causal Evidence for the Role of Neuronal Oscillations in Top–Down and Bottom–Up Attention. J. Cogn. Neurosci 2019, 31, 768–779. 30726180 10.1162/jocn_a_01376 PMC6701188

[49] Yuan Z; Chen H; Ding Z; Li Z; Song Y; Li X The Modulating Effect of Top-down Attention on the Optimal Pre-Target Onset Oscillatory States of Bottom-up Attention. Neuroscience 2021, 466, 186–195. 33865944 10.1016/j.neuroscience.2021.03.036

[50] Theves S; Chan JS; Naumer MJ; Kaiser J Improving Audio-Visual Temporal Perception through Training Enhances Beta-Band Activity. Neuroimage 2020, 206, 116312. 31669301 10.1016/j.neuroimage.2019.116312

[51] Viswanathan V; Bharadwaj HM; Heinz MG; Shinn-Cunningham BG Induced Alpha And Beta Electroencephalographic Rhythms Covary With Single-Trial Speech Intelligibility In Competition. Sci. Rep 2023, 13, 10216. 37353552 10.1038/s41598-023-37173-2 PMC10290148

[52] Price CN; Alain C; Bidelman GM Auditory-Frontal Channeling in and Bands Is Altered by Age-Related Hearing Loss and Relates to Speech Perception in Noise. Neuroscience 2019, 423, 18–28. 31705894 10.1016/j.neuroscience.2019.10.044 PMC6900454

[53] Obleser J; Weisz N Suppressed Alpha Oscillations Predict Intelligibility of Speech and Its Acoustic Details. Cereb. Cortex 2012, 22, 2466–2477. 22100354 10.1093/cercor/bhr325 PMC4705336

[54] Deng Y; Reinhart RMG; Choi I; Shinn-Cunningham B Causal Links between Parietal Alpha Activity and Spatial Auditory Attention. Elife 2019, 8, e51184. 31782732 10.7554/eLife.51184 PMC6904218

[55] Frey JN; Mainy N; Lachaux JP; Muller N; Bertrand O; Weisz N Selective Modulation of Auditory Cortical Alpha Activity in an Audiovisual Spatial Attention Task. J. Neurosci 2014, 34, 6634–6639. 24806688 10.1523/JNEUROSCI.4813-13.2014 PMC6608137

[56] Shinn-Cunningham BG; Best V Selective Attention in Normal and Impaired Hearing. Trends Amplif 2008, 12, 283–299. 18974202 10.1177/1084713808325306 PMC2700845

